# Optimal Maneuvering for Autonomous Vehicle Self-Localization

**DOI:** 10.3390/e24081169

**Published:** 2022-08-22

**Authors:** John L. McGuire, Yee Wei Law, Kutluyıl Doğançay, Sook-Ying Ho, Javaan Chahl

**Affiliations:** 1 UniSA STEM, University of South Australia, Mawson Lakes, SA 5095, Australia; 2 Joint and Operations Analysis Division, Defence Science and Technology Group, Melbourne, VIC 3207, Australia

**Keywords:** autonomous systems, optimal maneuvering, informative path planning, angle-of-arrival localization, information and sensor fusion, optimization and planning

## Abstract

We consider the problem of optimal maneuvering, where an autonomous vehicle, an unmanned aerial vehicle (UAV) for example, must maneuver to maximize or minimize an objective function. We consider a vehicle navigating in a Global Navigation Satellite System (GNSS)-denied environment that self-localizes in two dimensions using angle-of-arrival (AOA) measurements from stationary beacons at known locations. The objective of the vehicle is to travel along the path that minimizes its position and heading estimation error. This article presents an informative path planning (IPP) algorithm that (i) uses the determinant of the self-localization estimation error covariance matrix of an unscented Kalman filter as the objective function; (ii) applies an *l*-step look-ahead (LSLA) algorithm to determine the optimal heading for a constant-speed vehicle. The novel algorithm takes into account the kinematic constraints of the vehicle and the AOA means of measurement. We evaluate the performance of the algorithm in five scenarios involving stationary and mobile beacons and we find the estimation error approaches the lower bound for the estimator. The simulations show the vehicle maneuvers to locations that allow for minimum estimation uncertainty, even when beacon placement is not conducive to accurate estimation.

## 1. Introduction

Localization is the process of using acquired information to determine a vehicle’s position in a global frame [1]. Accurate localization is a prerequisite for safe UAV operations. A Global Navigation Satellite System (GNSS) such as the Global Positioning System is widely used in applications where the vehicle has lines of sight with navigation satellites. However, the signal might not be available when indoors; when the satellite signals are jammed; or when the vehicle lacks the hardware to process GNSS signals [1]. When GNSS is not a viable solution, visual information and alternate radio-frequency (RF) information can be used. A vehicle can self-localize by measuring relative ranges and bearings to objects that are at known locations in the environment. Computer vision algorithms detect visual landmarks from a camera image and determine the relative bearing between the camera and landmark. Bearing measurements can be combined with other sensor measurements, such as from Inertial Measurement Units (IMU) [2,3] or a laser range finder [4], to support self-localization. Audio signals can be used to self-localize by calculating the time-difference-of-arrival (TDOA) between measurements of a microphone array to estimate the angle-of-arrival (AOA) of sound sources in the environment [5]. The AOA of RF signals can be determined using phased antenna arrays, a mechanically steerable antenna, or multiple antennas [6].

Regardless of the measurement mode, *angle-based sensing* provides information on both position and orientation, enabling estimation of both position and orientation (pose) in two dimensions, unlike range-based sensing [7]. A minimum of three AOA measurements can be used to localize an autonomous vehicle in a known environment provided that the vehicle and beacons are not collinear or cocircular [8,9]. The work reported here focuses on AOA measurements for localization.

The problem of self-localization using beacon measurements can be framed as a wireless sensor network problem, where the objects at known locations are beacon nodes and the vehicle is a mobile node. Path planning in wireless sensor networks typically involves determining a path for a mobile beacon node [10,11]. The mobile beacon node has a method of accurately determining its location and then traverses a path to assist in the localization of other nodes in the network. The path planning scheme results in the mobile beacon node moving within the communication range of several other nodes to allow them to determine where they are using a localization algorithm [11]. The localization accuracy influences the network’s ability to achieve objectives. Similarly, the accuracy of a UAV self-localization scheme influences how well the UAV can perform tasks.

Recognizing that localization accuracy is dependent on measurement noise and vehicle/beacons geometry [12,13], our work uses the idea that a vehicle can minimize estimation uncertainty by traveling along a path that maximizes self-localization information. Typically when an autonomous vehicle maneuvers to complete a mission objective, the resultant vehicle/beacons geometry is suboptimal in terms of localization certainty. For example, the loitering-in-place maneuver of a fixed-wing UAV [14] would result in a suboptimal vehicle/beacons geometry. When an autonomous vehicle is awaiting tasking from a command station, there is an opportunity to allow the vehicle to undertake an information-gathering flight to improve its subsequent self-localization accuracy. This information-gathering maneuver is particularly useful for autonomous agents that hold ephemeral information and rely on receiving spatially distributed information to update an estimate.

Consider the problem of *optimal maneuvering* where a vehicle must determine its trajectory to minimize its position and heading uncertainty on a two-dimensional (2D) plane. The vehicle has some uncertainty about its initial position and knows the global position of several beacons in the environment. Using noisy bearing measurements received through its onboard sensors, the vehicle estimates its position and heading. The noise characteristics of the sensors are assumed to be known. The contribution of this article is an *l*-step look-ahead (LSLA) optimal maneuvering algorithm that determines the optimal heading for a vehicle self-localizing using AOA measurements. The algorithm uses an unscented Kalman filter (UKF) for state estimation. The output of the algorithm is an information-maximizing trajectory along which the vehicle will travel with minimal localization uncertainty. Simulations demonstrate the effectiveness of the proposed algorithm and the advantage of optimal maneuvering in various navigation scenarios.

The rest of this article is organized as follows. Section 2 discusses related work. Section 3 introduces a state-space model of the vehicle, including the measurement equation. Section 4 describes a framework for optimal maneuvering that employs an LSLA algorithm that minimizes a cost function to determine the optimal heading. Section 5 demonstrates the effectiveness of the algorithm in different scenarios in MATLAB Simulink simulations. Section 6 concludes the study.

## 2. Related Work

**Figure 1:**
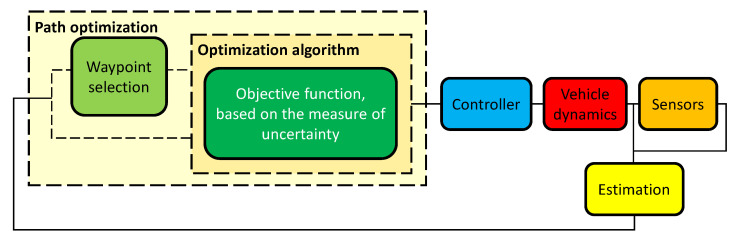
Block diagram of a generic IPP solution. Some solutions dispense with *waypoint selection*.

There are studies of *informative path planning* (IPP), which can be defined as the generation of a path or trajectory for a mobile sensor that maximizes the information gathered about its environment [15,16,17,18]. When formulated as an optimization problem, IPP comprises the key components depicted in Figure 1.

### 2.1. Measures of Uncertainty

The behavior of an IPP algorithm depends on the way the uncertainties in the information of interest are defined. In general, if the information of interest is modeled using a discrete random variable or discrete random vector, then the associated measure of uncertainty is available through discrete entropy. For continuous random variables or vectors, common uncertainty measures include differential entropy and the covariance matrix. Differential entropy is an extension of discrete entropy to continuous random variables/vectors and it takes on a value in [−∞,∞] [19,20]. Differential entropy is a relative measure for facilitating the comparison of the entropies of two continuous distributions. Below, we discuss the specific measures of uncertainty according to the types of IPP applications found in the literature.

In terrain monitoring, the aim is to minimize map uncertainty, which depends on the map representation and available sensing data [17]. One form of terrain monitoring is terrain texture classification, where a downward-facing camera is used to take images of the terrain. The map is separated into grid cells, and the probability of target (e.g., weed) occupancy is represented using an independent Bernoulli random variable; the associated measure of uncertainty is available through discrete entropy [17]. For modeling continuous environmental variables, Gaussian processes are commonly used [17]. The covariance of a Gaussian process, and in fact any stochastic process model, provides the associated measure of uncertainty. In spatial information gathering applications, information such as Wi-Fi signal strength can also be modeled with Gaussian processes [21,22], and accordingly, uncertainties can be expressed as covariance matrices.

In 3D building image reconstruction applications, IPP is about planning the path of an imaging drone to acquire images that can be used to accurately reconstruct 3D models of buildings. Schmid et al. [18] (Sec. IVA) defined information gain (inverse of uncertainty) as a sum of the “impact” of each sensed voxel. The impact of a view is influenced by uncertainties associated with image depth and state estimation. The uncertainty type is dependent on the algorithm used; for example, a single objective tree approach allows for a single objective function based on the uncertainty of an entire path [18]. The resultant IPP algorithm favors viewpoints that observe new areas and improve previously mapped sections.

Our work is a proposed solution to the IPP problem in the context of *self-localization*. Relevant to this context are the following topics in the area of *target localization*: optimal sensor-target geometry [13,23,24], stationary target localization [25,26,27], and dynamic target localization [16,28,29,30,31,32,33]. The aforementioned studies share the objective of determining sensor positions or trajectories that improve target localization performance. The key distinction between target localization and self-localization problems is that for self-localization, the estimation errors affect the sensing platform directly. This causes a compounding error that makes the estimation task more difficult. In many target localization problems, it is assumed that the sensing platform location is known precisely [13,16,28,31] and the estimation accuracy only affects the target location estimation.

The act of determining an optimal path is also found in applications such as missile guidance, for example in scenarios of target interception [34,35]. In these applications, it is often assumed that the onboard sensor suite is capable of detecting the target perfectly [34,35]. Because the target state is assumed to be perfectly known, the path planning can be determined using optimal control theory, for example, using Pontryagin’s maximum principle [33,34]. Pontryagin’s maximum principle has also been used to determine an optimal path for AOA tracking, however, under the assumption that the UAV’s coordinates are known exactly [33]. Because we assume that we do not have perfect state estimation capabilities, we opt to determine the optimal input by finding waypoints that maximize the informative objective function. In this article, the assumption is that the beacon locations are valid as often these beacons are stationary at known locations such as landmarks or radio towers.

### 2.2. Path Optimization

There are two main methods of path optimization. The first is to generate a set of potential waypoints for the vehicle and use an optimization algorithm to select sequential waypoints that produce the most informative path. The potential waypoints can be candidates for the vehicle’s next position or candidates for inclusion in the future trajectory of the vehicle. The second method performs optimization to decide an informative action (e.g., turning angle) based on admissible vehicle movements rather than considering existing waypoints.

#### 2.2.1. Waypoint Selection

A waypoint selection algorithm is used to generate potential waypoints for an IPP scheme to optimize over and determine an informative path. The waypoints must be generated such that they capture enough of the environment that informative trajectories can be determined, but sparse enough that the algorithms can be run in real time. A lattice grid search can be used to generate potential waypoints to perform measurements in 2D [16,21] and 3D [17] space. Tree-based algorithms including rapidly-exploring random tree (RRT) [36], rapidly-exploring information gathering (RIG) algorithm [22], reduced value iteration (RVI) [32], and their derivatives [18] are commonly used to determine paths for IPP problems. To expand the search tree, sampling procedures can be used [18] or points can be projected based on the dynamics of the mobile sensor given a finite set of control inputs [32]. As an alternative to using a grid of the physical map, an exhaustive search on a grid of the feasible control envelope can be used to find the optimal control inputs [37]. Once a set of potential waypoints are generated, an optimization strategy is needed to identify the most informative path.

#### 2.2.2. Optimization Algorithms

The path optimization problem is generally nonconvex and hence metaheuristic and numerical optimization algorithms are commonly used. Among these algorithms were greedy search (one-step look-ahead), reinforcement learning (RL), gradient descent, graph search methods, neural networks, and evolutionary algorithms such as the covariance matrix adaptation evolution strategy (CMA-ES).

Greedy search algorithms have been applied to IPP [37] and have been used as a baseline for performance assessment [21]. Greedy motion planning approaches are more efficient than look-ahead approaches at the expense of exploration range [37].

RL algorithms have a high initial computational complexity due to their learning-based nature. However, the execution time of RL algorithms increases linearly with budget and number of iterations rather than the exponential increase of a recursive greedy (RG) algorithm. Thus, for large maps, RL algorithms are more efficient than RG algorithms [21].

Methods of gradient descent use first or second-order finite difference approximations to determine the direction and hence choose the waypoint that minimizes the objective function [25,28,31,38]. Gradient descent methods converge to a local minimum and may not reach the global minimum, reducing their reliability [28].

Graph search methods are applicable whenever candidate trajectories can be represented as a graph. Hernandez [39] divided potential sensor locations into grids (a type of graph) and applied grid search to determine the optimal trajectory. A state space can also be discretized into a graph; for example, *state lattice* is a sampling of the state space in the form of a graph whose vertices are a discretized set of all reachable system states and whose edges are feasible controls [40]. A state-lattice-based trajectory can be computed by combining a finite set of feasible motion primitives. These motion primitives can be determined using numerical optimal control and describe the trajectory the vehicle takes between the positions on the lattice [16]. In the context of a mobile sensor performing IPP to track a mobile target, the state lattice of the *target* is of interest, and can be generated by calculating the (i) *a priori* target state distribution, (ii) sampling the distribution by taking the sigma points of the distribution to generate lattice states, (iii) connecting the matching sigma points to create candidate target trajectories [16]. For efficiency, heuristic graph search algorithms such as branch and bound [41] and algebraic redundancy-based pruning [42] can then be applied to determine the optimal trajectory.

Neural networks have been used to solve the optimal maneuvering problem [43]; however, they are not strictly optimizing, but rather classifying. They have been shown to run in a fraction of the time taken by a mathematical programming approach [43]. A downside of using neural networks is that they require a careful design of the network structure, and collecting a training dataset that leads to the desired behavior can be time- and resource-intensive.

The covariance matrix adaptation evolution strategy (CMA-ES) [44] is a global optimization routine based on evolutionary algorithms, and has been used to find an optimal path for an IPP problem [17]. Other evolutionary algorithms such as particle swarm optimization, genetic algorithm, and cuckoo search [45] have been used to solve the IPP problem. Evolutionary algorithms do not generally guarantee optimality and can suffer from slow convergence [46].

### 2.3. Objective Function

The objective function is defined such that the utility or information gain along the vehicle trajectory is maximized, or equivalently, the uncertainty along the trajectory is minimized [17]. Not all measures of uncertainty discussed above can be used as is in the formulation of an objective function because some measures are not scalar whereas an objective function has to be. This explains the existence of the D-optimality and A-optimality criteria.

The D-optimality criterion maximizes the determinant of the *Fisher information matrix* (FIM), resulting in the minimization of the volume of the estimation error ellipsoid [47]. The D-optimality criterion has been used to determine maneuvers and thereby target-observer geometries that maximize estimation accuracy for bearings-only localization of a stationary target [25] and a moving target [28,45,48]. The D-optimality criterion has also been used to generate paths for mobile beacons to improve the self-localization accuracy of a UAV they are in communication with [49]. For IPP problems that involve maximizing a receiver’s self-localization accuracy, the D-optimality criterion has been shown to produce the lowest RMSE compared to when other optimality criteria are used [37].

The A-optimality criterion maximizes the decrease in the error covariance matrix trace rather than the inverse of the determinant of the covariance matrix because the latter is computationally expensive [17]. The A-optimality criterion has been used to determine optimal flight paths for multiple UAVs using AOA measurements to localize targets in 2D [31,38,39,43,50] and 3D [51,52].

An advantage of the D-optimality criterion over the A-optimality criterion is that the former does not depend on the scale of the estimated variables [53]. This advantage is relevant as the vehicle states are position and heading. Different measurement units produce different trajectories for the A-optimality criterion but not necessarily for the D-optimality criterion.

### 2.4. Comparison of the Proposed Algorithm with Existing Literature

Our proposed approach is a waypoint selection algorithm where an exhaustive search is used to determine the optimal path. The novelty in our approach is the waypoint selection algorithm; it is based on constant-speed vehicle kinematics and allows for the trajectory to be determined over a planning horizon. The waypoints are selected based on the admissible motion of the vehicle, producing achievable locations at each time step of the planning horizon, with an accuracy defined by the process noise and estimation error. The end result is an informative path planned to maximize the effectiveness of the self-localization algorithm. One of the novelties of this work is the context. Target localization is the focus of many research works, however, the ability of a system to perform self-localization is inherently implied due to the abundant use of GNSS localization systems. For operations in GNSS-denied environments, the ability to self-localize is of the utmost importance. Furthermore, the ability to plan a path to maximize the informativeness of the sensor measurements is a process that is ignored in many scenarios as they assume perfect knowledge of vehicle position. The informative path planning algorithm presented in this article is a precursor to a framework wherein a vehicle can maneuver to minimize its estimation uncertainty to then enhance any target localization operation (or indeed any other application that requires accurate location and heading estimation) subsequently enacted.The waypoints generated by a sampling method [17,22] may not be reachable due to the kinematic constraints of the vehicle. The vehicle may take several epochs to arrive at the desired waypoint within a satisfactory margin. Our application is also novel; optimal maneuvering or IPP algorithms for the self-localization of an autonomous vehicle using AOA measurements have not been presented in the literature to the best of the author’s knowledge.

Our proposed approach shares similarities with other waypoint selection algorithms that are dependent on the dynamics or the kinematics of a vehicle [37,39]. The vehicle used in [39] has variable speed, and the waypoint selection algorithm analyzes subsequently smaller subsections of the feasible travel area until the objective function is minimized or the maximum number of iterations is reached. The waypoint selection algorithm presented in [37] assumes a variable speed vehicle, with constraints on the maximum vehicle velocity and acceleration, whereas we have assumed constant speed. The similarities to our approach include that the optimal solutions for the information-based objective functions are found by gridding the control feasibility region and an exhaustive search is used to select the optimal waypoint. Our approach also grids the control feasibility region, however, due to our single control input, this results in a line, rather than an arc. Unlike our approach, the waypoint selection algorithm in [37] only looks one step ahead. We consider a constant-speed vehicle as it simplifies the optimization problem, and is relevant to the application under consideration where the vehicle is operating at a cruise velocity. By considering a constant speed, the search area at each time step is reduced, reducing the computational complexity and allowing for a larger planning horizon.

We compare the proposed algorithm to the RIG-tree algorithm developed by Hollinger and Sukhatme [22]. Their algorithm randomly samples points in the configuration space to select potential waypoints. For each new point that is sampled, the algorithm extends branches from existing nodes that are within a distance $r_{near}$ to the newest node. The RIG algorithm has been used as a benchmark IPP algorithm in several studies [15,17]. It can be modified to form a fixed-horizon version that allows for incremental replanning and a better comparison to our proposed method. The algorithm has been adapted for compatibility with our application, by using the objective function defined in Equation (11).

## 3. Problem Setup

Table 1 defines the frequently used symbols in the ensuing discussion.

We consider a two-dimensional optimal maneuvering problem in an environment with $n_{b}\geq 3$ location beacons (also called “anchor nodes” in the sensor network literature). Onboard sensors read a noisy AOA measurement to each beacon. The vehicle moves with a fixed forward speed *v* and the control input is the rotational rate of the vehicle ω. The following discrete-time nonlinear state equation describes the simplified kinematics of the system:

(1) $$\left[\begin{array}{c} x_{v,k+1}\\ y_{v,k+1}\\ \phi_{k+1} \end{array}\right]=\left[\begin{array}{c} x_{v,k}+TV\cos\left(\phi_{k}+\frac{T\omega_{k}}{2}\right)\\ y_{v,k}+TV\sin\left(\phi_{k}+\frac{T\omega_{k}}{2}\right)\\ \phi_{k}+T\omega_{k} \end{array}\right]+w_{k}.$$

Above, *T* is the sample period. Let *n* denote the number of states, and the n×1 state vector for the vehicle kinematics is given by

(2) $$x_{k}={\left[\begin{array}{ccc} x_{v,k} & y_{v,k} & \phi_{k} \end{array}\right]}^{T}.$$

where ${\left[x_{v,k}\;y_{v,k}\right]}^{T}$, $\left[\phi_{k}\right]$ are the vehicle’s position and heading, respectively, at time *k*. In Equation (1), *T* is the sample period, *V* is the constant vehicle speed and $\omega_{k}$ is the rotational vehicle speed. $w_{k}$ is the process noise that accounts for unknown perturbations of the system states, and is modeled as a zero-mean white Gaussian noise with covariance $Q_{k}$:

(3) $$w_{k}∼N\left(0,Q_{k}\right).$$

$Q_{k}$ is the n×n process noise covariance matrix and is modeled as a diagonal matrix with elements $\left(q_{x}T^{2}/2,q_{y}T^{2}/2,q_{\phi }T^{2}/2\right)$. For AOA localization, the nonlinear measurement equation is

(4) $$z_{k}=h\left(x_{k}\right)+v_{k}.$$

Above, $z_{k}$ is a vector of $n_{b}$ AOA measurements, $v_{k}$ is the measurement noise, modeled as zero-mean white Gaussian noise with covariance $R_{k}\left(x_{k}\right)$:

(5) $$v_{k}∼N\left(0,R_{k}\left(x_{k}\right)\right).$$

The *i*th element of the vector $h(x_{k})$ is given by

(6) $$h_{i}\left(x_{k}\right)=∠\left({\left[x_{bi},y_{bi}\right]}^{T}-{\left[x_{v,k},y_{v,k}\right]}^{T}\right)-\phi_{k}.$$

**Figure 2:**
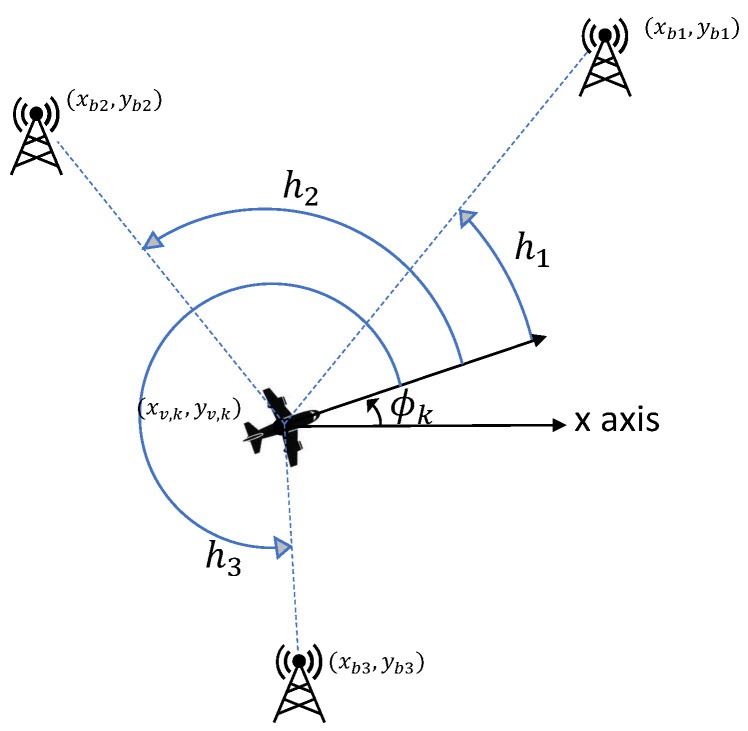
Angle-of-Arrival measurements from three beacons.

Above, ∠· denotes the angle of a vector. While $h_{i}$ is the true angle between the *i*th beacon and the vehicle’s heading, as depicted in Figure 2, $z_{i}$ is a noisy measurement of $h_{i}$. The covariance matrix of the AOA measurement noise $R_{k}\left(x_{k}\right)$ is a diagonal $n_{b}\times n_{b}$ matrix with the (i,i) element given by

(7) $$r_{i}\left(x_{k}\right)=\sigma_{i}^{2}d_{i,k}^{2}+\sigma_{i,min}^{2}.$$

Above, $\sigma_{i}^{2}$ is the signal noise variance for the *i*th measurement at unit distance from the beacon; $\sigma_{i,min}^{2}$ is the floor noise variance for the sensor reading; $d_{i,k}$ is the distance between the vehicle and the *i*th beacon at time *k*. The noise variance for AOA measurements is dependent on the sensor characteristics. Self-localization is achieved through estimation of the state vector defined in Equation (2).

## 4. Optimal Maneuvering

Under the proposed scheme, a vehicle navigates along a trajectory that it determines on the fly. The trajectory is intended to be optimal in terms of position and heading estimation error. To determine the next waypoint, a vehicle solves an optimization problem, the objective function of which is related to the information content of the sensor measurements, subject to some trajectory constraints. The optimization result gives the next waypoint, based on which the control input, angular speed $\omega_{k}$, is determined.

The subsequent subsections discuss state estimation using the unscented Kalman filter (see Section 4.1), information content and objective function modeling using Fisher information (see Section 4.2), optimization constraints in terms of trajectory constraints (see Section 4.3), and finally determination of the optimal waypoint and angular speed (see Section 4.4).

### 4.1. State Estimation

The Kalman filter produces estimates of the states of a linear dynamic system using a series of noisy measurements observed over time [54]. The standard Kalman filter does not provide accurate estimates when the state transition equation and measurement equation are nonlinear. The *unscented Kalman filter* (UKF) is a linear estimator that is applicable to nonlinear systems [55]. The key to the UKF is the *unscented transform*, which (i) deterministically approximates the state vector with 2n+1 *sigma points*; (ii) transforms the sigma points using the nonlinear state function; (iii) uses the mean and covariance of the transformed sigma points to predict the future mean and covariance of the state vector. Let ${\hat{x}}_{k|k}$ be the n×1*a posteriori* state estimate at time step *k*. At time step k+1, the unscented transform is applied to ${\hat{x}}_{k|k}$ to produce ${\hat{x}}_{k+1|k}$, the *a priori* state estimate for time step k+1. The following state update equations are then applicable:

(8a) $$\begin{array}{cc} {\hat{x}}_{k+1|k+1} & ={\hat{x}}_{k+1|k}+K_{k+1}\left(z_{k+1}-{\hat{z}}_{k+1|k}\right). \end{array}$$

(8b) $$\begin{array}{cc} P_{k+1|k+1} & =P_{k+1|k}-K_{k+1}P_{vv,k+1|k}K_{k+1}^{T}. \end{array}$$

(8c) $$\begin{array}{cc} K_{k+1} & =P_{xv,k+1|k}P_{vv,k+1|k}^{-1}. \end{array}$$

Above, $P_{k+1|k}$ is the n×n*a priori* error covariance matrix for ${\hat{x}}_{k+1|k}$. ${\hat{z}}_{k+1|k}$ is the $n_{b}\times 1$ *a priori* measurement estimate produced by applying the unscented transform to ${\hat{z}}_{k|k}$. $P_{vv,k+1|k}$ is the $n_{b}\times n_{b}$*a priori* innovation covariance matrix. $P_{xv,k+1|k}$ is the $n\times n_{b}$ *a priori* cross correlation matrix. ${\hat{x}}_{k+1|k+1}$ and $P_{k+1|k+1}$ are the resultant n×1*a posteriori* state estimate and n×n error covariance matrix, respectively, at the end of time step k+1. $K_{k+1}$ is the $n\times n_{b}$ Kalman gain. Upon obtaining a posterior state estimate, the optimal maneuvering scheme determines the next waypoint by finding one that maximizes the information content of the sensor measurements.

### 4.2. Information Content and Objective Function

Fisher information is a way of measuring the information that observable random variables x have about unknown variables θ over the probability p(x|θ) [56]. In the optimal maneuvering problem, the observable random variables are the AOA measurements, and the unknown variables are the vehicle’s position and heading. In a Bayesian estimation problem, the *Bayesian FIM* indicates how much information the data contains about the estimated variables. The Bayesian FIM is based on the log-likelihood of the joint probability p(x,θ), denoted L(x,θ)[57]. Since L(x,θ) is equivalent to L(x|θ)+L(θ), the Bayesian FIM consists of two additive terms, namely the information from the data and the *a priori* information [58,59,60], i.e.,

(9) $${\left[J_{B}\right]}_{i,j}=E\left\{\frac{\partial L(x|\theta )}{\partial \theta_{i}}\frac{\partial L(x|\theta )}{\partial \theta_{j}}\right\}+E\left\{\frac{\partial L(\theta )}{\partial \theta_{i}}\frac{\partial L(\theta )}{\partial \theta_{j}}\right\}.$$

Above, ${\left[J_{B}\right]}_{i,j}$ is the {ij}th entry of the Bayesian FIM, $\theta_{i}$ is the *i*th element of θ, $\theta_{j}$ is the *j*th element of θ, and E{Z} is the expectation of *Z*. The *posterior Cramér-Rao lower bound* (PCRLB [59], or equivalently the Bayesian CRB [58] or Van Trees CRB [60]) gives the lower bound for the error covariance of a Bayesian estimator and is equal to the inverse of the Bayesian FIM [58,61]. The relationship between the error covariance matrix of the Bayesian estimator, the PCRLB, and the Bayesian FIM are given by [58,59]:

(10) $$E\left\{\left[g\left(x\right)-\theta \right]{\left[g\left(x\right)-\theta \right]}^{T}\right\}⪰C_{\mathrm{PCRLB}}=J_{B}^{-1}.$$

Above, g(x) is a function of x that gives an estimate of θ, $C_{\mathrm{PCRLB}}$ is the PCRLB, and $J_{B}$ is the Bayesian Fisher information matrix in Equation (9). In Equation (10), the expression A⪰B means the matrix A−B is positive semi-definite.

The error covariance matrix of the UKF can be approximated by the PCRLB [23]. Therefore, the inverse of the error covariance matrix $P_{k|k}^{-1}$ is approximately equal to the Bayesian FIM. An objective function that minimizes the determinant of the UKF error covariance matrix will maximize the determinant of the Bayesian FIM and therefore minimize the self-localization estimation error. Such an objective function is consistent with the D-optimality criterion (see Section 2). Denote by $J_{k}$ the objective function at time *k*, then

(11) $$J_{k}=\left|P_{k+1|k}\right|.$$

A crucial observation is that in Equation (11), the predicted error covariance matrix $P_{k+1|k}$ rather than the posterior error covariance matrix $P_{k+1|k+1}$ is used, because at time *k*, the measurement at time k+1 is not yet available, and hence $P_{k+1|k+1}$ is not yet known. Using the D-optimality criterion, the optimal maneuvering algorithm selects waypoints that minimize the volume of the error ellipsoid for the position and heading estimates. However, not all of these waypoints are applicable because the vehicle, like any real-world vehicle, is subject to motion/trajectory constraints, as explained in the next subsection.

### 4.3. Motion and Trajectory Constraints

In Equation (1), the rotational control of the vehicle is governed by the linear state transition

(12) $$\phi_{k+1}=\phi_{k}+T\omega_{k}.$$

Above, $\phi_{k}$ is the vehicle’s heading at time *k*, $\omega_{k}$ is the control input at time *k*, and *T* is the sample period. The optimal maneuvering scheme detailed in Section 4.4 determines a desired heading $\theta_{d}$ and subsequently a control input such that Equation (12) becomes

(13) $$\phi_{k+1}=\theta_{d}.$$

The vehicle’s movement is constrained by three main physical limitations of the system:

**Forward speed constraint** Given a fixed forward speed *V*,
  (14) $$||{\left[\begin{array}{c} x_{v,k+1}-x_{v,k}\\ y_{v,k+1}-y_{v,k} \end{array}\right]||}_{2}=VT.$$
  Above, $||\cdot {\left|\right|}_{2}$ denotes the Euclidean norm.
**Turn-rate constraint** Physical limitations of any real-world vehicle constrain the vehicle to a maximum turn rate, denoted by $\omega_{\max}$. Change in heading between time steps *k* and k+1 is limited by
  (15) $$|\phi_{k+1}-\phi_{k}|\leq T\omega_{\max}.$$
**Proximity constraints to the beacons** Proximity constraints arise from the need to prevent numerical instability which occurs when the vehicle gets too close to a beacon. $n_{b}$ proximity constraints are implemented as radii from the beacons according to the inequalities
  (16) $$||{\left[\begin{array}{c} x_{v,k+1}-x_{bi}\\ y_{v,k+1}-y_{bi} \end{array}\right]||}_{2}\geq d_{\min},\;\mathrm{for}\;i=1,\cdots ,n_{b}.$$
  Above, $d_{\min}$ is the minimum allowable distance between the vehicle and beacon, $\left(x_{bi},y_{bi}\right)$ is the position of the *i*th beacon, and $\left(x_{v,k+1},y_{v,k+1}\right)$ is the vehicle position at time step k+1.

The optimal maneuvering algorithm takes the constraints above into account when determining the desired heading. Satisfying the speed and turn-rate constraints in Equations (14) and (15) restricts the vehicle trajectory such that at time *k*, the possible vehicle positions at time k+1 are defined by an arc with radius VT centered at $\left(x_{v,k},y_{v,k}\right)$. The state update can be defined using the following:

(17a) $$\begin{array}{cc} \theta_{\max} & =\frac{T\omega_{\max}}{2}. \end{array}$$

(17b) $$\left[\begin{array}{c} x_{v,k+1}\\ y_{v,k+1} \end{array}\right]=\left[\begin{array}{c} x_{v,k}\\ y_{v,k} \end{array}\right]+VT\left[\begin{array}{c} \cos(\phi_{k}+\theta_{\mathrm{arc}})\\ \sin(\phi_{k}+\theta_{\mathrm{arc}}) \end{array}\right].$$

Above, $-\theta_{\max}\leq \theta_{\mathrm{arc}}\leq \theta_{\max}$. The objective function value at time step k+1 can be estimated by projecting the vehicle position to a point on the arc defined in Equation (17) and using the UKF predicted error covariance matrix as per Equation (11). This process forms the basis for the selection of an optimal waypoint detailed in the next subsection.

### 4.4. Optimal Waypoint Selection

An optimal waypoint is determined by evaluating the objective function $J_{k}$ defined in Equation (11) at each point on the arc defined in Equation (17), and selecting the point that minimizes $J_{k}$. To limit the number of evaluations, the arc between $-\theta_{\max}$ and $\theta_{\max}$ is discretized into *m* points, i.e.,

$$\theta_{\mathrm{arc}}\in \left\{-\theta_{\max},\left(-1+\frac{2}{M-1}\right)\theta_{\max},\cdots ,\theta_{\max}\right\}.$$

**Figure 3:**
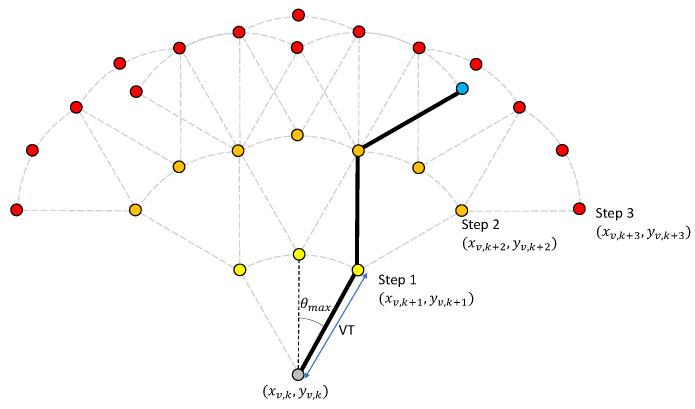
An example of a tree-like search space used by the optimal maneuvering algorithm. Here, m=3 and l=3. Suppose evaluation of $J_{k}$ along the thick branch gives the lowest value, then the thick branch is the *optimal branch*.

The value of *m* is chosen based on a trade-off between decision resolution and computational efficiency. Multi-step planning provides a larger search area by determining waypoints several time steps in advance; we call these steps *look-ahead steps*. For each point on the arc at time k+1, another arc can be calculated, representing the candidate waypoints at time k+2. This branching process can be extended for an arbitrary number of look-ahead steps. Denote by *l* the number of look-ahead steps, then *m* points on each arc and *l* steps of looking ahead amount to a search area of $m^{l}$ candidate waypoints (see Figure 3), and l×m objective function evaluations at each look-ahead step. Thus, low computational complexity favors a small *l*.

Another consideration that favors a small *l* is that the UKF state estimate cannot be updated when predicting future costs as future measurements are unknown and the accuracy of objective function prediction drops as *l* increases due to the compounding of unmodeled disturbances. The *optimal branch* is defined as the set of waypoints from the first to the *l*th look-ahead step that minimizes the value of $J_{k}$ (see Figure 3). Denoted by $\theta_{\mathrm{arc}}^{*}$ the first waypoint on the optimal branch, then the desired heading $\theta_{d}$ is derived from $\theta_{\mathrm{arc}}^{*}$ as $\theta_{d}=\theta_{\mathrm{arc}}^{*}+\phi_{k}$ The control input in Equation (12) becomes $\omega_{k}=\frac{\theta_{\mathrm{arc}}^{*}}{T}$. If any of the candidate waypoints violate the proximity constraints in Equation (16), they are discarded. If no waypoints satisfy the constraint, the algorithm steers the vehicle away from the beacon.

## 5. Simulation Results

We present simulations to evaluate the performance of the optimal maneuvering algorithm detailed in Section 4.The simulations were performed in MATLAB Simulink. Five scenarios were simulated to demonstrate the performance of the algorithm when (i) different values of the parameters *m* and *l* are used; (ii) mobile beacons are used; (iii) communication with some of the beacons is disrupted; (iv) stationary beacons are positioned far away from the initial position of the vehicle; (v) a less informative beacon formation is used. In every scenario, there were three beacons in the environment. The waypoint selection component of the RIG algorithm [22] was used for comparison to evaluate performance.

In each scenario, 100 simulation runs were performed. The actual objective function was calculated by first calculating the true error covariance matrix using the data from all simulation runs $\left|\mathrm{cov}(x_{k}-{\hat{x}}_{k|k})\right|$. The objective function is calculated using the statistics from the 100 simulation runs and, as a result, is itself indicative of the statistics of the performance of the estimation. By calculating the error covariance matrix of the 100 simulation runs, we are able to analyze the true performance of the algorithm. The reason we distinguish between the objective function calculated using all of the simulation data and that determined using the error covariance matrix produced by the UKF in the individual simulations is that the UKF error covariance matrix depends on the estimate of the system state, as the informative of the sensor measurements is location-dependant. The theorized objective function value may be different than the true value resulting from the self-localization errors.The metrics used to evaluate the performance of the optimal maneuvering algorithms were the average value and the settling time of the objective function. For calculating the settling time, we used $\log_{10}$ of the objective function due to the range of computed values spanning many orders of magnitude.

### 5.1. Comparison of the LSLA Algorithm for Different Values of l and m

Table 2 and Table 3 present scenario one, where mobile beacons were implemented. Three mobile beacons were randomly placed in a 20 × 20 km area centered around the origin. The mobile beacons moved at a speed of 50 m/s along straight paths towards randomized locations within the environment for the duration of the simulation. The parameters used in the simulations are as follows: $\sigma_{i}^{2}=6.8\times 10^{-6}$, $\sigma_{i,min}^{2}=3.04\times 10^{-4}$, $\left[q_{x},q_{y},q_{\theta }\right]=\left[10^{-4},10^{-4},10^{-4}\right]$, V=30 m/s, $\omega_{\max}=0.1$ rad/s, T=1 s, simulation time = 500 s, $d_{\min}=0.1$ km, $x_{0}={\left[0,0,\pi \right]}^{T}$, and $P_{0}=\mathrm{diag}\left(2.25,2.25,0.0076\right)$.

Table 2 and Table 3 present the average objective function value and settling time, respectively, for different LSLA *l* and *m* values. Analyzing the rows in Table 2, the minimum objective function value was achieved when m=7 for all *l* values. Analyzing the columns of Table 2, the minimum objective function was achieved in three cases when l=3 and once when l=4 and l=2. Most simulations where l=3 outperformed those where l=4, possibly due to the decrease in the accuracy of calculating the UKF error covariance matrix at the $l^{th}$ step as *l* is increased (see Section 4.4). Analyzing the settling time in Table 3, there is no obvious link between *m* and a low settling time. However, l=3 is the best choice for reducing the average objective function value and the settling time.

### 5.2. Comparison between LSLA and RIG Algorithms

**Figure 4:**
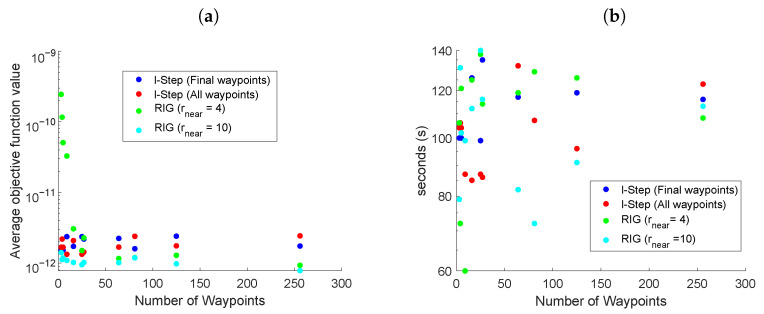
(**a**) The average value of the objective function for the last 200 s of simulation using the LSLA and RIG algorithms with moving beacons. (**b**) Settling time of the objective function.

Figure 4 presents scenario two, comparing the LSLA and RIG algorithms for different numbers of waypoints, in a 10 × 10 km environment with mobile beacons. Two variants of LSLA were compared (i) when all the waypoints in the tree were evaluated; (ii) when only waypoints at the *l*th step were evaluated. The RIG algorithms with values of 4km and 10 km for $r_{near}$ were investigated. The value of $r_{near}$ was large so that the RIG covered a sufficiently large search area at the expense of computational complexity.

In Figure 4a, the RIG variant using $r_{near}=4$ achieved an objective function value of approximately two orders of magnitude larger than the other algorithms for a small number of samples. This is due to the RIG algorithm not being able to build up a sufficiently large map, when $r_{near}$ is increased to 10, this issue is avoided and the RIG algorithm exclusively outperforms the others, but it should be noted that this greatly increases the computational complexity of the algorithm (see Section 5.6). Comparing LSLA when all waypoints were evaluated with when only the *l*th step waypoints were evaluated, neither one clearly outperformed the other with respect to an average objective function value, however, the settling time was, in general, (Figure 4b) better when all of the waypoints were considered. The benefit of evaluating all of the waypoints is that short-term maneuvers can be considered, at the expense of slightly more computational complexity.

### 5.3. Mobile Beacons and Beacon Communication Loss

**Figure 5:**
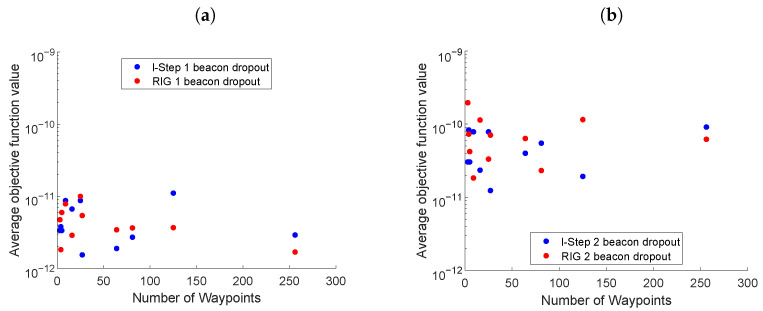
The average value of the objective function for the last 200 s of simulation using the LSLA and RIG algorithms after communications with (**a**) one beacon and (**b**) two beacons are dropped.

Figure 5 presents the third scenario, where beacons were mobile and after 200 s, communications were disrupted between the vehicle and one (Figure 5a) or two (Figure 5b) beacons. The simulation parameters were the same as in Figure 4, LSLA evaluated all waypoints, l=3, and m=3. The RIG algorithm parameter $r_{near}=10$ and the number of waypoints was 81. Comparing the average objective function values in Figure 5, with those in the case where there are no beacon communication disruptions in Figure 4, both the LSLA and RIG algorithms were able to maintain an informative trajectory in the presence of disrupted beacon communications.

### 5.4. Stationary Beacons Far from Initial Vehicle Position

**Figure 6:**
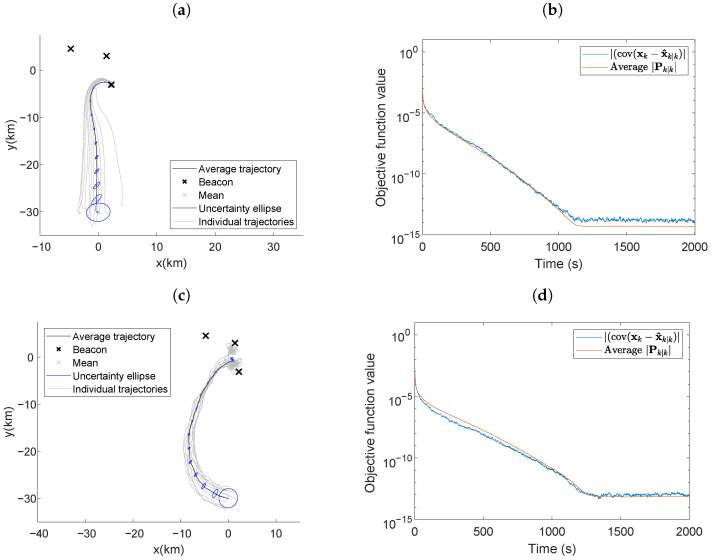
Average vehicle trajectory with position uncertainty ellipses placed every 100 s using (**a**) LSLA and (**c**) RIG algorithms in the optimal maneuvering scheme. Actual and average theoretical objective function value using (**b**) LSLA and (**d**) RIG algorithms.

Figure 6 presents scenario four, with stationary beacons placed at locations (2.200,−3.084), (1.374,3.006), and (−4.750,4.552). The simulation parameters that differed from those in scenario one were: $\sigma_{i}^{2}=6.8\times 10^{-7}$, $\sigma_{i,min}^{2}=7.615\times 10^{-5}$, $q_{x},q_{y},q_{\theta }=\left[10^{-5},10^{-5},10^{-5}\right]$, simulation time = 2000s, and $x_{0}={\left[0,-30,\pi \right]}^{T}$. In a real-world scenario, it is unlikely that fixed beacons would be in a favorable geometry, motivating the placement of three beacons in a cluster far from the vehicle’s initial position. The average trajectory over the 100 simulations was plotted with the one-standard-deviation error ellipses for positions placed at 100-second intervals. Ten individual trajectories are plotted alongside the average trajectory plot for reference.

The D-optimality criterion minimizes the area of the error ellipsoid for the estimates. Figure 6a,c display the average trajectory of the vehicle when minimizing self-localization estimation error, indicating the vehicle exhibited a source-seeking behavior. The source-seeking behavior is due to the D-optimality criterion for self-localization using AOA measurements being inversely dependent on the distances between the vehicle and the beacons [12]. The vehicle in Figure 6a only approximates the one beacon because LSLA does not steer the vehicle close enough to other beacons.

When using the LSLA, the vehicle traveled towards the center of the beacons along a slightly curved trajectory and then travels towards the closest beacon. The individual trajectories were slightly different but adhered to this general observation and are presented in Figure 6a. Figure 6b shows that the actual value of $\left|\mathrm{cov}(x_{k}-{\hat{x}}_{k|k})\right|$ reaches a similar value to the average determinant of the error covariance matrix produced by the UKF and the settling time was 954 s. When using the RIG algorithm, the vehicle trajectory was more curved, resulting in a settling time of 1098 s in Figure 6d. LSLA achieved a smaller average objective function value for the last 500 s of the simulation than the RIG algorithm with $6.78\times 10^{-14}$ and $1.00\times 10^{-13}$, respectively. The objective function value is directly related to the self-localization performance as detailed in Section 4.2. As the vehicle maneuvered along an informative path, the vehicle received more informative AOA measurements that improved the estimation performance and reduced the objective function value.

### 5.5. Optimal Maneuvering with an Undesirable Vehicle/Beacons Geometry

**Figure 7:**
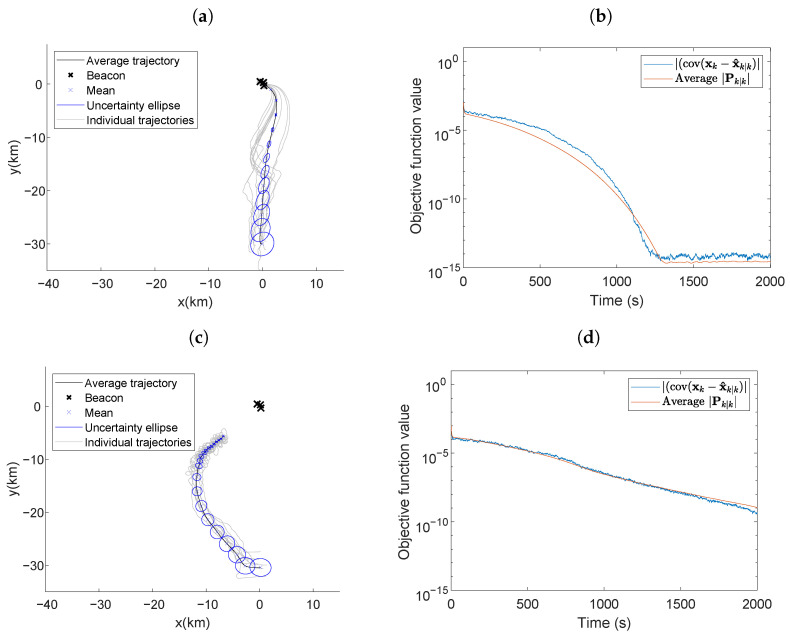
Average vehicle trajectory with position uncertainty ellipses placed every 100 s using (**a**) LSLA and (**c**) RIG algorithms in the optimal maneuvering scheme with a less informative beacon formation. Actual and average theoretical objective function value using (**b**) LSLA and (**d**) RIG algorithms.

Figure 7 presents scenario five, where all parameters were the same as those used in Figure 6 except the beacons were placed at locations (0.220,−0.308), (0.137,0.301), and (−0.475,0.455). The close placement of the beacons produced a vehicle/beacons geometry that led to less informative AOA measurements at a long range. The average trajectory in Figure 7a has the same characteristics as observed in Figure 6a; the vehicle traveled towards the center of the beacons along a curved trajectory and proceeded to loiter around the beacons. The individual trajectories in Figure 7a were not as direct as those in Figure 6a; the most informative path often required meandering. Due to the close beacon proximity, the individual trajectories maneuvered around all three beacons instead of circling one. When the RIG algorithm was used in this environment, the vehicle’s self-localization performance was worse. The vehicle’s average trajectory was a curved path towards the beacons. However, the individual trajectories were even less direct than when using LSLA. The indirect trajectories caused the vehicle to not arrive close to the beacon in the simulation time and since the measurement information is related to the inverse of the distance from the beacons, the objective function value was several orders of magnitude larger at the end of the simulation time when compared to when the vehicle used the LSLA in Figure 6b.

### 5.6. Computational Complexity

**Figure 8:**
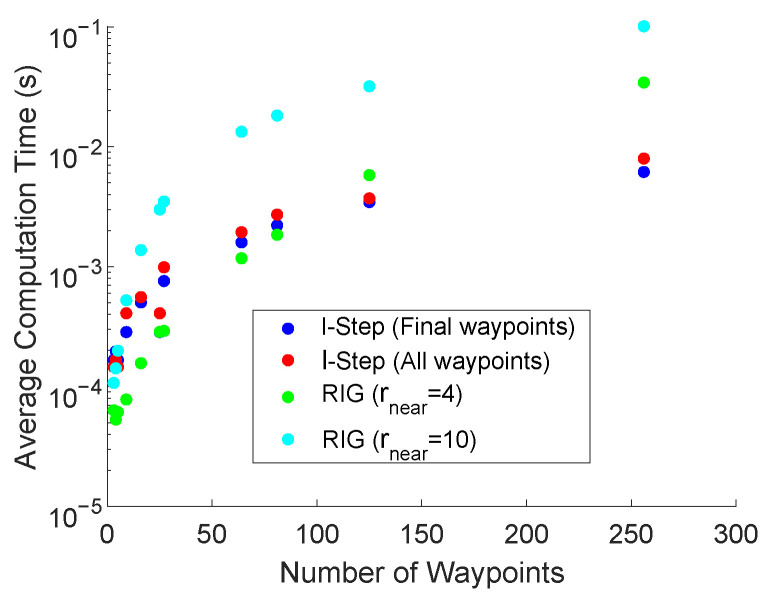
The average time for the LSLA and RIG algorithms to produce an optimal waypoint as the total number of waypoints is increased.

Figure 8 presents the average computation time of the LSLA and RIG algorithms used in scenario two in Figure 4. For both methods, the computational requirement of calculating the objective function value for each node is the same, the computational complexity of generating the error covariance matrix of the unscented Kalman filter is [62] a polynomial function dependent on the number of states and the number of measurements. In our implementation, it does not grow with the length of the trajectory.

The computational complexity of the RIG algorithm is limited by the calculation of near nodes which searches the list of available nodes. In the worst-case scenario, where all available waypoints need to be extended in each iteration of the algorithm, this is an $O(n^{2})$ operation. Checking if a potential node should be pruned also involves searching across all existing nodes. The memory requirements for the RIG algorithm grow linearly in the number of nodes as the stored information remains constant [22].

The time complexity of the LSLA to generate the potential waypoints is minimal as for each iteration, the waypoints are fixed relative to the vehicle position. Defining the search space is an O(n) operation. The memory requirement for LSLA grows linearly in the number of nodes.

## 6. Conclusions

The *l*-step look-ahead optimal maneuvering algorithm leads the vehicle towards a location that minimizes estimation error. We evaluated the algorithms for different values of *l* and *m* corresponding to the number of steps and resolution of each step, respectively. We found that increasing *l* (the number of steps) is generally more beneficial for reducing the objective function value and the settling time than increasing the value of *m*. We compared our algorithm to the RIG-tree [22] informative path planning algorithm in scenarios with mobile beacons, beacon communication dropout, and undesirable beacon initial positions, and found that while our algorithm was comparable in performance to the RIG algorithm in most scenarios, it performed better in environments with less information available as it was able to maneuver along a more direct path to gather information. This is most clearly seen in the scenario where the beacons are in a small formation and located far away from the initial location of the vehicle in Figure 7. The objective function for the vehicle that selects waypoints using the RIG-tree algorithm does not reach a minimum value in the duration of the simulation, unlike when LSLA is used.

Future work involves equipping the vehicle with the ability to predict the trajectory of mobile beacons in order to determine improved maneuvers when loitering in the presence of mobile beacons. The optimal maneuvering framework that is presented can be extended to form a multi-objective optimization problem that balances the information gathering action with achieving other mission objectives. Another avenue of future work is to conduct experiments to validate our algorithm on real hardware. An updated simulation environment will be developed to benchmark the performance of the algorithm on hardware. The simulation would require changes to the model and sensor characteristics to more closely align with the physical system. 

## Figures and Tables

**Table 1:** List of frequently used symbols and their definitions.

Symbols	Definitions
$x_{v,k}$, $y_{v,k}$, $\phi_{k}$	Vehicle’s 2D coordinates and heading at time *k*
$z_{k}$	AOA measurement vector at time *k*
*V*	Vehicle’s forward speed
*T*	Sample period
$\omega_{k}$	Vehicle’s rotational speed at time *k*
*n*, $n_{b}$	Number of states and beacons
$x_{bi}$, $y_{bi}$	2D coordinates of the *i*th beacon
$J_{k}$	Objective function defined in Equation (11)
*m*	Number of candidate waypoints in one time step
*l*	Number of look-ahead steps when determining the optimal waypoint

**Table 2:** Average objective function value for different *m* and *l*. Results are in the order of $10^{-11}$.

	m = 3	m = 4	m = 5	m = 6	m = 7
l = 1	2.89	2.73	3.27	2.97	2.71
l = 2	2.60	2.50	2.98	2.97	2.48
l = 3	3.07	3.02	2.80	2.62	2.22
l = 4	3.26	2.48	2.81	2.77	2.42

**Table 3:** Settling time of the objective function in seconds for different values of *m* and *l*.

	m = 3	m = 4	m = 5	m = 6	m = 7
l = 1	184	190	200	194	214
l = 2	187	164	214	230	174
l = 3	166	188	165	173	171
l = 4	183	201	173	170	185

## Data Availability

The data presented in this study are available on request from the corresponding author.

## References

[1] Bulusu N., Heidemann J., Estrin D. (2000). GPS-less low-cost outdoor localization for very small devices. IEEE Pers. Commun..

[2] Caballero F., Merino L., Ferruz J., Ollero A. (2009). Vision-based odometry and SLAM for medium and high altitude flying UAVs. J. Intell. Robot. Syst..

[3] Langelaan J., Rock S. Passive GPS-free navigation for small UAVs. Proceedings of the 2005 IEEE Aerospace Conference.

[4] Loevsky I., Shimshoni I. (2010). Reliable and efficient landmark-based localization for mobile robots. Robot. Auton. Syst..

[5] Ogiso S., Kawagishi T., Mizutani K., Wakatsuki N., Zempo K. (2015). Self-localization method for mobile robot using acoustic beacons. ROBOMECH J..

[6] Jais M., Ehkan P., Ahmad R., Ismail I., Sabapathy T., Jusoh M. Review of angle of arrival (AOA) estimations through received signal strength indication (RSSI) for wireless sensors network (WSN). Proceedings of the 2015 International Conference on Computer, Communications, and Control Technology (I4CT).

[7] Niculescu D., Nath B. Ad hoc positioning system (APS) using AOA. Proceedings of the IEEE INFOCOM 2003. Twenty-second Annual Joint Conference of the IEEE Computer and Communications Societies (IEEE Cat. No.03CH37428).

[8] Betke M., Gurvits L. (1997). Mobile robot localization using landmarks. IEEE Trans. Robot. Autom..

[9] Esteves J.S., Carvalho A., Couto C. Generalized geometric triangulation algorithm for mobile robot absolute self-localization. Proceedings of the 2003 IEEE International Symposium on Industrial Electronics (Cat. No.03TH8692).

[10] Lin Y., Tao H., Tu Y., Liu T. (2019). A node self-localization algorithm with a mobile anchor node in underwater acoustic sensor networks. IEEE Access.

[11] Sabale K., Mini S. (2019). Anchor node path planning for localization in wireless sensor networks. Wirel. Netw..

[12] McGuire J., Law Y.W., Chahl J., Doğançay K. (2021). Optimal Beacon Placement for Self-Localization Using Three Beacon Bearings. Symmetry.

[13] Bishop A.N., Fidan B., Anderson B.D., Doğançay K., Pathirana P.N. (2010). Optimality analysis of sensor-target localization geometries. Automatica.

[14] Peliti P., Rosa L., Oriolo G., Vendittelli M. (2012). Vision-based loitering over a target for a fixed-wing UAV. IFAC Proc. Vol..

[15] Boström-Rost P., Axehill D., Hendeby G. (2018). On Global Optimization for Informative Path Planning. IEEE Control Syst. Lett..

[16] Boström-Rost P., Axehill D., Hendeby G. Informative Path Planning for Active Tracking of Agile Targets. Proceedings of the 2019 IEEE Aerospace Conference.

[17] Popović M., Vidal-Calleja T., Hitz G., Chung J.J., Sa I., Siegwart R., Nieto J. (2020). An informative path planning framework for UAV-based terrain monitoring. Auton. Robot..

[18] Schmid L., Pantic M., Khanna R., Ott L., Siegwart R., Nieto J. (2020). An Efficient Sampling-Based Method for Online Informative Path Planning in Unknown Environments. IEEE Robot. Autom. Lett..

[19] Huber M.F., Bailey T., Durrant-Whyte H., Hanebeck U.D. On entropy approximation for Gaussian mixture random vectors. Proceedings of the 2008 IEEE International Conference on Multisensor Fusion and Integration for Intelligent Systems.

[20] Michalowicz J.V., Nichols J.M., Bucholtz F. (2013). Handbook of Differential Entropy.

[21] Wei Y., Zheng R. Informative Path Planning for Mobile Sensing with Reinforcement Learning. Proceedings of the IEEE INFOCOM 2020-IEEE Conference on Computer Communications.

[22] Hollinger G.A., Sukhatme G.S. (2014). Sampling-based robotic information gathering algorithms. Int. J. Robot. Res..

[23] Doğançay K., Hmam H. (2008). Optimal angular sensor separation for AOA localization. Signal Process..

[24] Cheng Y., Wang X., Morelande M., Moran B. (2013). Information geometry of target tracking sensor networks. Inf. Fusion.

[25] Oshman Y., Davidson P. (1999). Optimization of observer trajectories for bearings-only target localization. IEEE Trans. Aerosp. Electron. Syst..

[26] Hoffmann G.M., Tomlin C.J. (2010). Mobile sensor network control using mutual information methods and particle filters. IEEE Trans. Autom. Control.

[27] Grocholsky B., Makarenko A., Durrant-Whyte H. Information-theoretic coordinated control of multiple sensor platforms. Proceedings of the 2003 IEEE International Conference on Robotics and Automation (Cat. No.03CH37422).

[28] Passerieux J.M., Van Cappel D. (1998). Optimal observer maneuver for bearings-only tracking. IEEE Trans. Aerosp. Electron. Syst..

[29] Martínez S., Bullo F. (2006). Optimal sensor placement and motion coordination for target tracking. Automatica.

[30] Bishop A.N., Pathirana P.N. (2008). Optimal Trajectories for Homing Navigation with Bearing Measurements. IFAC Proc. Vol..

[31] Doğançay K. Single- and multi-platform constrained sensor path optimization for angle-of-arrival target tracking. Proceedings of the 2010 18th European Signal Processing Conference.

[32] Schlotfeldt B., Thakur D., Atanasov N., Kumar V., Pappas G.J. (2018). Anytime Planning for Decentralized Multirobot Active Information Gathering. IEEE Robot. Autom. Lett..

[33] Andreev K.V., Rubinovich E.Y. (2016). Moving observer trajectory control by angular measurements in tracking problem. Autom. Remote Control.

[34] Galyaev A.A., Lysenko P.V., Rubinovich E.Y. (2021). Optimal stochastic control in the interception problem of a randomly tacking vehicle. Mathematics.

[35] Zhang H., Zhang Y., Zhang P. (2021). Optimal guidance law for intercepting the active defense aircraft with terminal angle constraint. J. Phys. Conf. Ser..

[36] Papachristos C., Khattak S., Alexis K. Uncertainty-aware receding horizon exploration and mapping using aerial robots. Proceedings of the 2017 IEEE International Conference on Robotics and Automation (ICRA).

[37] Kassas Z.M., Arapostathis A., Humphreys T.E. (2015). Greedy Motion Planning for Simultaneous Signal Landscape Mapping and Receiver Localization. IEEE J. Sel. Top. Signal Process..

[38] Xu S., Doğançay K., Hmam H. Distributed path optimization of multiple UAVs for AOA target localization. Proceedings of the 2016 IEEE International Conference on Acoustics, Speech and Signal Processing (ICASSP).

[39] Hernandez M.L. Optimal sensor trajectories in bearings-only tracking. Proceedings of the Seventh International Conference on Information Fusion.

[40] Pivtoraiko M., Knepper R.A., Kelly A. (2009). Differentially constrained mobile robot motion planning in state lattices. J. Field Robot..

[41] Papadimitriou C.H., Steiglitz K. (1998). Combinatorial Optimization: Algorithms and Complexity.

[42] Vitus M.P., Zhang W., Abate A., Hu J., Tomlin C.J. (2012). On efficient sensor scheduling for linear dynamical systems. Automatica.

[43] Wang W., Bai P., Zhou Y., Liang X., Wang Y. (2019). Optimal configuration analysis of AOA localization and optimal heading angles generation method for UAV swarms. IEEE Access.

[44] Hansen N., Lozano J.A., Larrañaga P., Inza I., Bengoetxea E. (2006). The CMA Evolution Strategy: A Comparing Review. Towards a New Evolutionary Computation: Advances in the Estimation of Distribution Algorithms.

[45] Sabet M., Fathi A., Daniali H.M. (2016). Optimal design of the own ship maneuver in the bearing-only target motion analysis problem using a heuristically supervised extended Kalman filter. Ocean Eng..

[46] Patle B., Pandey A., Parhi D., Jagadeesh A. (2019). A review: On path planning strategies for navigation of mobile robot. Def. Technol..

[47] Ucinski D. (2000). Optimal sensor location for parameter estimation of distributed processes. Int. J. Control.

[48] Zhang H., Dufour F., Anselmi J., Laneuville D., Nègre A. Piecewise optimal trajectories of observer for bearings-only tracking of maneuvering target. Proceedings of the 2018 IEEE Aerospace Conference.

[49] McGuire J., Law Y.W., Chahl J. Mobile beacon path planning for optimal unmanned aerial vehicle self-localization. Proceedings of the AIAC 2021: 19th Australian International Aerospace Congress. Engineers Australia.

[50] Roh H., Cho M.H., Tahk M.J. Trajectory optimization using Cramér-Rao lower bound for bearings-only target tracking. Proceedings of the 2018 AIAA Guidance, Navigation, and Control Conference.

[51] Moreno-Salinas D., Pascoal A., Aranda J. (2013). Sensor networks for optimal target localization with bearings-only measurements in constrained three-dimensional scenarios. Sensors.

[52] Xu S., Doğançay K. (2017). Optimal sensor placement for 3-D angle-of-arrival target localization. IEEE Trans. Aerosp. Electron. Syst..

[53] Ucinski D. (2004). Optimal Measurement Methods for Distributed Parameter System Identification.

[54] Kalman R.E. (1960). A new approach to linear filtering and prediction problems. J. Basic Eng..

[55] Julier S.J., Uhlmann J.K. New extension of the Kalman filter to nonlinear systems. Proceedings of the Signal Processing, Sensor Fusion, and Target Recognition VI.

[56] Kay S.M. (1993). Fundamentals of Statistical Signal Processing.

[57] Niu R., Vempaty A., Varshney P.K. (2018). Received-signal-strength-based localization in wireless sensor networks. Proc. IEEE.

[58] Spagnolini U. (2018). Statistical Signal Processing in Engineering.

[59] Tichavsky P., Muravchik C.H., Nehorai A. (1998). Posterior Cramér-Rao bounds for discrete-time nonlinear filtering. IEEE Trans. Signal Process..

[60] Van Trees H.L. (2004). Detection, Estimation, and Modulation Theory, Part I: Detection, Estimation, and Linear Modulation Theory.

[61] Zuo L., Niu R., Varshney P.K. (2010). Conditional posterior Cramér–Rao lower bounds for nonlinear sequential Bayesian estimation. IEEE Trans. Signal Process..

[62] Lu S., Cai L., Ding L., Chen J. Two Efficient Implementation Forms of Unscented Kalman Filter. Proceedings of the 2007 IEEE International Conference on Control and Automation.

